# Assessing the Accuracy of Lateral Cephalogram in Quantifying Three-Dimensional Pharyngeal Airway Morphology Compared to Cone-Beam Computed Tomography

**DOI:** 10.7759/cureus.57301

**Published:** 2024-03-31

**Authors:** Syed Ashique Abdulhameed, Mohamed Abdulcader Riyaz SS, Mohammed Almutairy, Salman Khan N, Saikarthik Jayakumar, Prachi Gaonkar

**Affiliations:** 1 Department of Orthodontics and Dentofacial Orthopaedics, Meenakshi Ammal Dental Vollege and Hospital, Chennai, IND; 2 Department of Maxillofacial Surgery and Diagnostic Science, College of Dentistry, Qassim University, Ar Rass, SAU; 3 Department of Oral Medicine and Periodontology, College of Dentistry, Qassim University, Ar Rass, SAU; 4 Department of Orthodontics and Dentofacial Orthopaedics, Pushpagiri College of Dental Sciences, Thiruvalla, IND; 5 Department of Maxillofacial Surgery and Diagnostic Science, College of Dentistry, Majmaah University, Al Majmaah, SAU; 6 Department of Orthodontics and Dentofacial Orthopaedics, Terna Dental College and Hospital, Mumbai, IND

**Keywords:** malocclusion, orthodontics, cbct, upper and lower pharyngeal space, lateral cephalogram

## Abstract

Background: When it comes to orthodontic diagnosis and treatment planning, the structures of the upper and lower airway space are crucial because of the role they play in craniofacial development.

Aim: The major objective of this study was to evaluate the accuracy of lateral cephalogram in the evaluation of upper and lower pharyngeal space by comparing it to clinical usage of cone-beam computed tomography (CBCT) in quantifying the 3D morphology of the pharyngeal airway.

Methods and materials: In total, 70 patients were included in the study. They had both a CBCT scan and a lateral cephalogram performed within a week of each other. Different cephalometric landmarks have been utilized to estimate linear and area dimensions for use in lateral cephalogram airway investigations. By superimposing the lateral cephalogram measurement of the vertical height of the pharyngeal airway over axial CBCT slices of 0.8 to 1 mm in thickness, airway volumes were calculated. For this study, we measured the pharyngeal airway space in each patient in two dimensions (2D) using the airway area from the lateral cephalogram and in three dimensions (3D) using the airway volume from the CBCT scan over the same region of interest, using a uniform scale and magnification throughout all split 3D volumes.

Results: The mean value of the area of pharyngeal space calculated by lateral cephalograph analysis (LCA) was 336.35 ± 86.49 mm^2^. The maximum value was 551.234 mm^2^. The minimum value was 206.32 mm^2^. The mean value of the volume of the same area calculated using CBCT was 3409.11 ± 1237.96 mm^3^. The maximum value was 5887.23 mm^3^. When the area calculated using LCA was compared with the volume calculated using CBCT, the correlation between them was significant statistically (r=0.831, p-value =0.000). The mean values of volume evaluated in 3D CBCT in males were 4198±1008 mm^3^ while for females it was 2980±1134.5 mm^3^. During the statistical analysis, these observations were found to have a positive correlation with increased volume of pharyngeal space in males as compared to that of females (p=0.006). The values of the area of pharyngeal space calculated using LCA in males was 370.1±60.9 mm^2.^ while it was 301.9±88 mm^2 ^ in females.

Conclusion: The area estimated for the pharyngeal airway on LCA correlates strongly with the volume determined by a CBCT scan. Since we have considered pharyngeal space analysis using CBCT to be a reliable and standard methodology, therefore a positive correlation of area calculated using LCA with volume calculated using CBCT shows that the analysis made by LCA can be reliable.

## Introduction

Airway function has significant effects on neuromuscular adaptations, nasopharyngeal blockage, development, growth, respiration, and speech [1,2]. The size of the upper airway is also thought to have a role in obstructive sleep apnea. This is especially important for teenagers who are displaying abnormal facial symptoms and skeletal deformities [3,4]. Researchers have shown that many cases of malocclusion may be traced back to problems with breathing, a condition known as "adenoid face." Various skeletal patterns have been explored in other investigations of the upper airway [5,6].

The consequences of airway obstructions on dentition, speech, and craniofacial development are best studied via experimental investigations, even if airway obstructions may resolve spontaneously over time. In light of this, orthodontists need reliable diagnostic instruments that accurately educate both the orthodontist and any other medical professionals who may need to be consulted [7,8]. The orthodontist only does limited, subjective assessments of potential airway abnormalities, often based on a lateral cephalogram [9,10]. However, the diagnostic value of this kind of airway anatomy examination is limited. Distortion, variations in magnification, and the superimposition of the bilateral craniofacial components are only some of the problems that arise when trying to portray a three-dimensional structure in a two-dimensional format [11-14].

When it comes to orthodontic diagnosis and treatment planning, the structures of the upper airway space (UAS) are crucial because of the vital role they play in craniofacial development [12-14]. In addition, breathing disorders like obstructive sleep apnea might have their roots in craniofacial morphologic aspects, particularly the anatomy of the upper airway. The research we have done so far shows that obstructive sleep apnea (OSA) is linked to smaller UAS dimensions. Using cone-beam CT (CBCT), some authors found that patients with OSA had a decreased lateral dimension and a smaller cross-sectional area of the upper and lower pharyngeal space compared to snorers [9]. In another study, researchers found that a considerable narrowing of the posterior airway space was linked to the return of OSA in previously treated teenagers [10]. Adenoids, soft palate length, and tongue dimensions are some of the other UAS soft tissue features that have been proposed as important morphological factors in OSA [11, 12].

Using lateral cephalograms, many studies have looked at whether or not upper pharyngeal space size is linked to other craniofacial traits [13-14], computed tomography [15], or a CBCT scan [16-24] in those who are otherwise healthy, without any pharyngeal or breathing problems. The association between maxillary and mandibular pharyngeal space dimensions and sagittal skeletal pattern is controversial, with varying findings reported. Sagittal skeletal malocclusion has been proven to impact pharyngeal space size in various studies [18]. Others have not been able to show any correlation [19, 20]. Variables may account for the differences shown in the aforementioned research (e.g. sample age [12] gender [13, 14], ethnicity [15], nasal cavity, oral cavity, and hypopharynx) as well as clashing due to correlated elements such as vertical and horizontal growth patterns and measuring area [16], categorization based on skeleton vs. teeth, requirements for inclusion (overweight and smoking), etc. [17], accuracy and proficiency in detecting cephalometric landmarks in clinical settings, linear, angle, ratio, area, and volume measures, computed tomography (CT) against traditional lateral cephalograms [18], head position during imaging, and manual versus computer digital tracing [22, 23].

The lateral cephalogram radiograph has no volume or cross-sectional area measurements, which is a major drawback. The use of CBCT technology as a diagnostic tool in the examination of airways has recently gained traction. The raw data is used to recreate the 3D object; therefore, zooming in is not a problem [15-18]. CBCT produces an isotropic picture, allowing for precise and anatomically correct linear and angular measurements. Orthodontic patients are not commonly referred for CBCT testing since the technology is not as widely accessible as conventional radiography [19, 20]. The major objective of this study was to evaluate the accuracy of the lateral cephalogram in the evaluation of upper and lower pharyngeal space by comparing it to the clinical usage of CBCT in quantifying the 3D morphology of the pharyngeal airway. We have considered pharyngeal space analysis using CBCT to be a reliable and standard methodology.

## Materials and methods

All young adults referred to an imaging center for a lateral cephalogram and CBCT scans over eight months were included in this retrospective cross-sectional investigation. Patients were included in the study if they met the inclusion criteria and had both a CBCT scan and a lateral cephalogram performed within a week of each other. The present inquiry focuses on CBCT scans that were performed while the patient was sitting up to prevent any changes to the airway space that may have resulted from lying down. Evaluation of the temporomandibular joint and impacted teeth were the most prevalent indications for CBCT referral. Participants were not allowed to wear bite splints, have a history of craniofacial anomalies or orthognathic surgery, or be less than 20 years of age.

This research employed a convenient sample size and its power was evaluated after the fact, using the value of the correlation coefficient at the 0.05 significance level. After contacting 196 possible volunteers, we only identified 70 who met the requirements. The sample size was 70. Most participants were rejected since the time between their two picture shoots was more than a week. Cranex D (Soredex, Helsinki, Finland) was used to capture the lateral 2D cephalograms at KVP = 70, mA = 10, and magnification = 9.8. After being photographed, the resolution of the scanned cephalograms is between 150 and 300 dpi. A NewTomVGi CT scanner (Quantitative Radiology, Verona, Italy) with a maximum field of view (FOV) of 115 was used to collect all of the CBCT volume scans.

**Figure 1:**
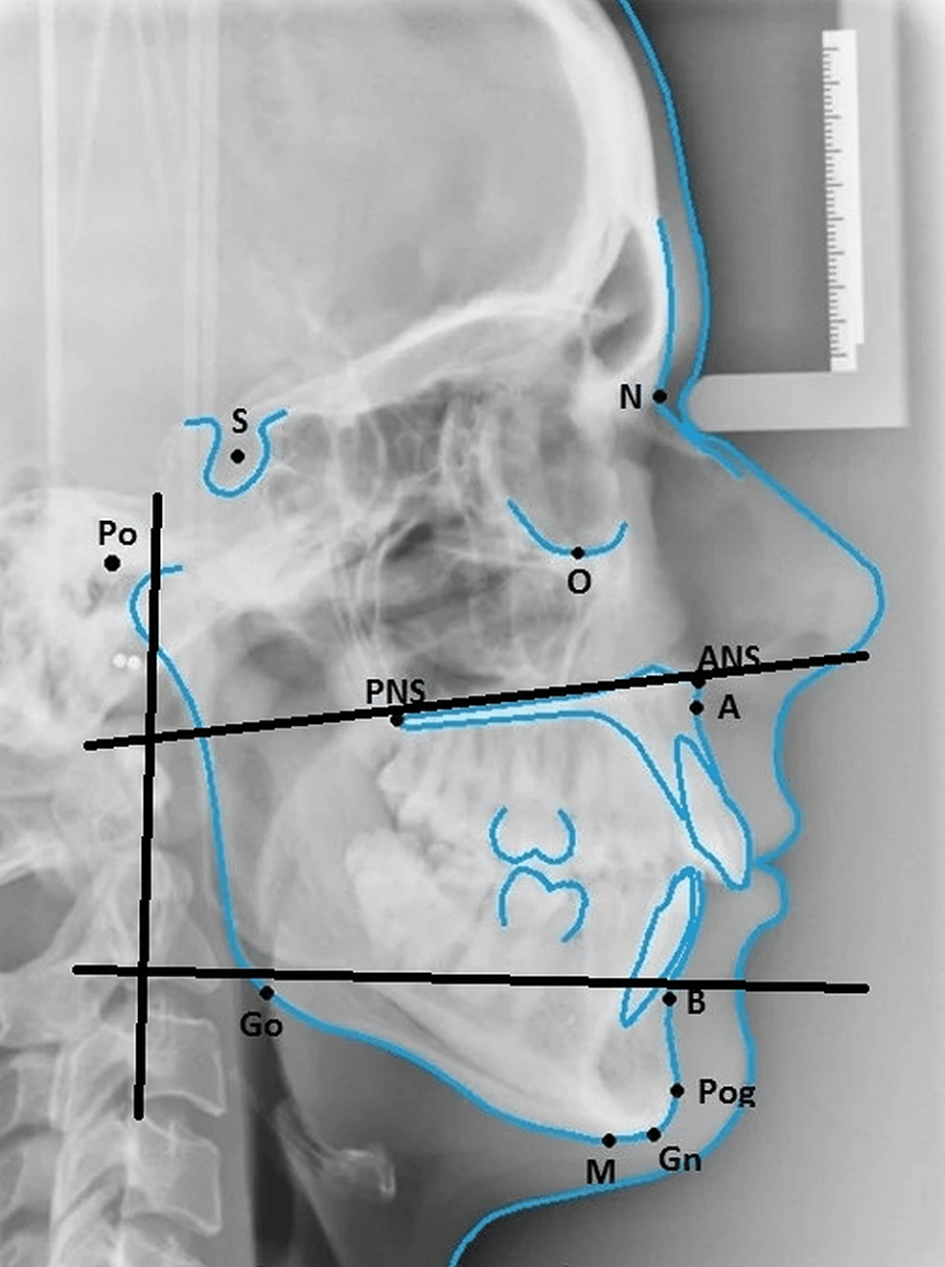
Lateral cephalograph showing the lines used dor the analysis

Different cephalometric landmarks have been utilized to estimate linear and area dimensions for use in lateral cephalogram airway investigations. The following morphological characteristics of all patients' pharyngeal airways, as seen on a lateral cephalogram were recorded: Line 1 runs from the rear of the tongue to the front of the soft palate (an ANS to PNS extension), while line 2 runs from the tip of the epiglottis to the floor of the mouth. Third, the horizontal plane bisects the diagonal (1/2) from the point of no return (PNS) to the lower boundary (Figure 1).

**Figure 2:**
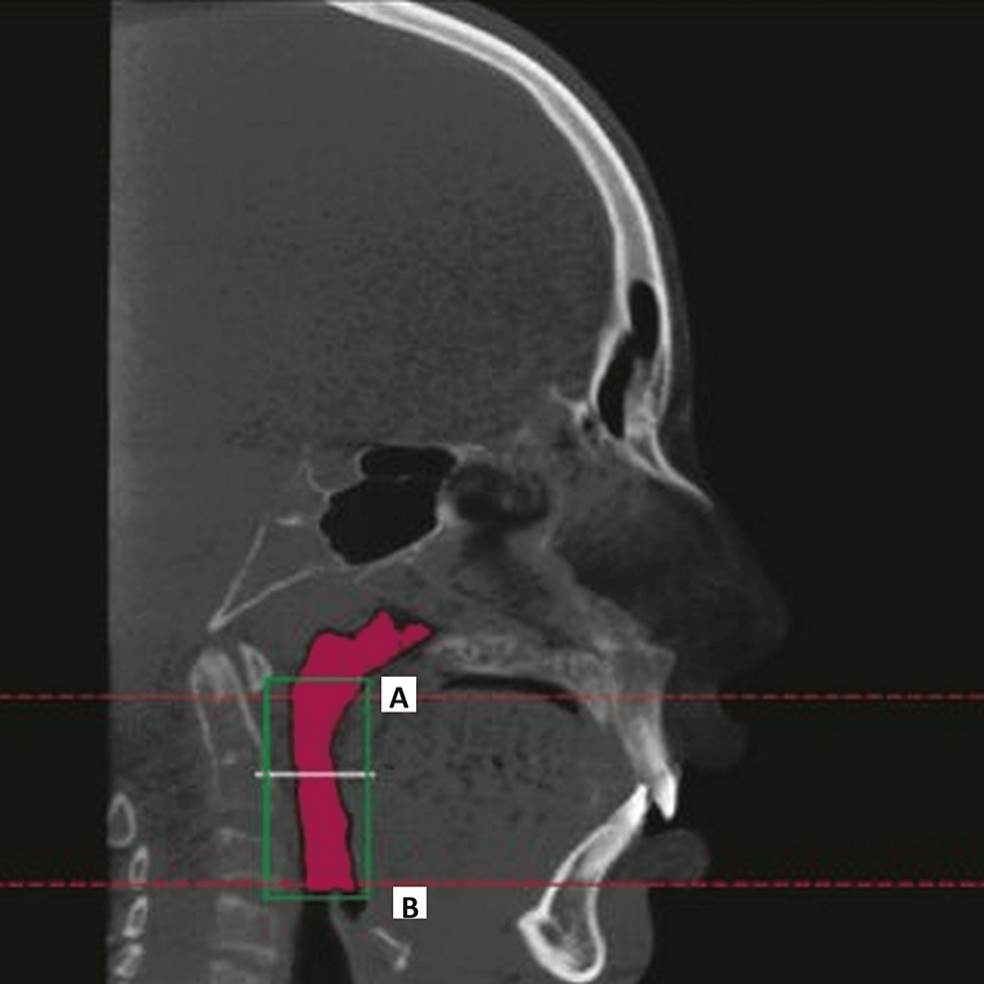
CBCT depicting the pharyngeal space with the area of interest A: superior limit B: inferior limit

To quantify airway volume along the same anatomical limits, the same planes were imported into the 3D image. The superior and inferior limits of the area of interest are visible in a sagittal cross-section across the middle of the sagittal plane. (Figure 2).

**Figure 3:**
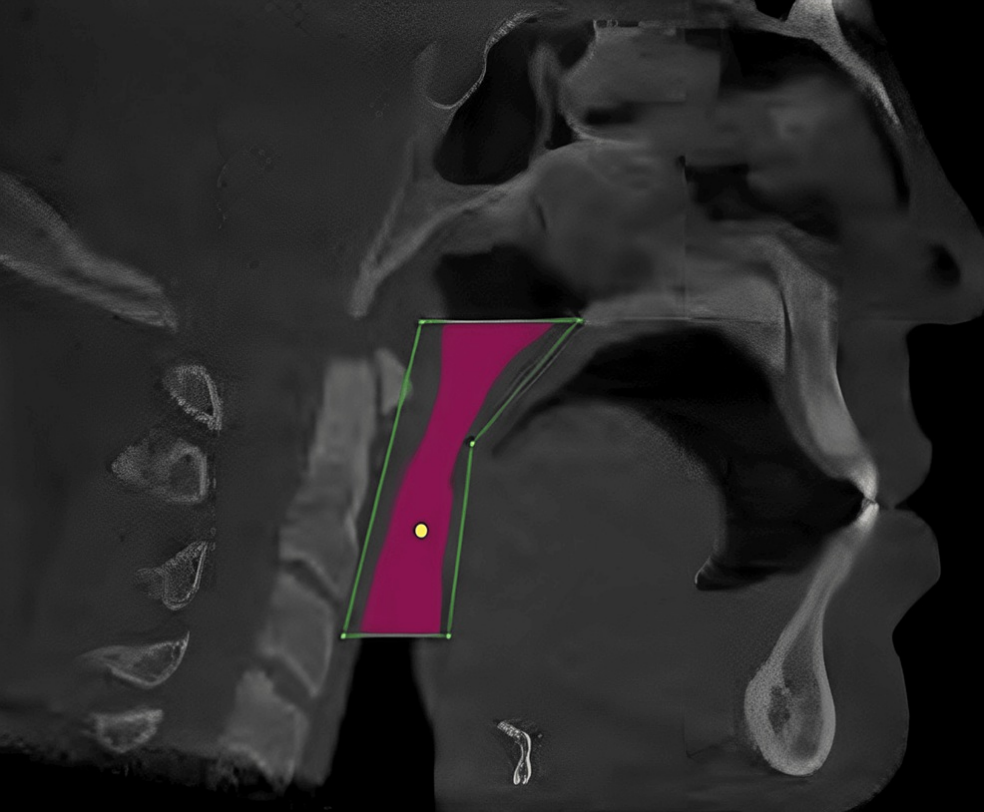
Image of the cone-beam CT depicting the pharyngeal space

Axial, coronal, The CBCT axial reconstruction plane was reoriented relative to the patient's coronal and sagittal reference planes. By superimposing the lateral cephalogram measurement of the vertical height of the pharyngeal airway over axial CBCT slices of 0.8 to 1 mm in thickness, airway volumes were calculated. For this study, we measured the pharyngeal airway space in each patient in two dimensions (2D) using the airway area from the lateral cephalogram and in three dimensions (3D) using the airway volume from the CBCT scan over the same region of interest, using a uniform scale and magnification throughout all split 3D volumes. We have considered pharyngeal space analysis using CBCT to be a reliable and standard methodology; therefore, the correlation of area calculated using LCA with volume calculated using CBCT was evaluated to find out if the analysis made by LCA was reliable (Figure 3).

We estimated the bivariate correlation coefficient (r) and used the Pearson correlation coefficient test to see whether the two variables were statistically related. The Mann-Whitney U test was used to examine if there were statistically significant differences between the sexes on 2D and 3D measurements. All statistical analyses were performed in SPSS version 18 (Chicago, USA).

## Results

In this study, 70 study participants were evaluated. It included 46 males and 24 females. The mean age of study participants was 22.85 ± 2.74 years (Table 1).

**Table 1:** Demographic details of study participants

Gender	
Male	46
Female	24
Mean age	22.85 ± 2.74 years

The mean value of the area of pharyngeal space calculated by lateral cephalograph analysis (LCA) was 336.35 ± 86.49 mm^2^. The maximum value was 551.234 mm^2^. The minimum value was 206.32 mm^2^. The mean value of the volume of the same area calculated using CBCT was 3409.11 ± 1237.96 mm^3^. The maximum value was 5887.23 mm^3^. When the area calculated using LCA was compared with the volume calculated using CBCT the correlation between them was significant statistically (r=0.831, p-value=0.000). Since we have considered pharyngeal space analysis using CBCT to be a reliable and standard methodology, therefore, the positive correlation of area calculated using LCA with volume calculated using CBCT shows the analysis made by LCA can be reliable (Table 2).

**Table 2:** Descriptive information on airway volume as well as area

Measurements	Area mm^2^ (LCA)	Volume mm^3^ (CBCT analysis)
n	70	70
Mean± SD	336.35 ± 86.49	3409.11 ± 1237.96
Max	551.234	5887.23
Min	206.32	1846.34
r value	0.831	
P value	0.001	

The mean values of volume evaluated in 3D CBCT in males were 4198±1008 mm^3^ while it was 2980±1134.5 mm^3^. The observations were found to have a positive correlation on carrying out statistical analysis with increased volume of pharyngeal space in males as compared to that of females (p=0.006). The values of the area of pharyngeal space calculated using LCA in males was 370.1±60.9 mm^2^ while it was 301.9±88 mm^2^ in females. The observations were found to have a positive correlation on carrying out statistical analysis with increased area of pharyngeal space in males as compared to that of females (p=0.005) (Table 3).

**Table 3:** Comparison of three-dimensional measurements and two-dimensional pharyngeal airway measurements between different sexes

Variable	Male	Female	P value
Volume in CBCT 3D (mm^3^)	4198±1008	2980±1134.5	0.006
Area in Cephalogram 2D (mm^2^)	370.1±60.9	301.9±88	0.005

## Discussion

The majority of prior airway research has relied on morphologic or functional methods to determine airway constriction and function [15, 22-25]. In orthodontics, evaluating the upper airway has traditionally been done using a lateral cephalogram, with the use of landmarks that help define the airway [25]. The major objective of this study was to evaluate the accuracy of a lateral cephalogram in comparison to the clinical usage of CBCT in quantifying the 3D morphology of the pharyngeal airway.

In this research, the mean value of the area of pharyngeal space calculated by LCA was 336.35 ± 86.49 mm^2^. The maximum value was 551.234 mm^2^. The minimum value was 206.32 mm^2^. The mean volume of the same area calculated using CBCT was 3409.11 ± 1237.96 mm^3^. The maximum value was 5887.23 mm^3^. When the area calculated using LCA was compared with the volume calculated using CBCT, the correlation between them was significant statistically. Since we have considered pharyngeal space analysis using CBCT to be a reliable and standard methodology, a positive correlation of area calculated using LCA with volume calculated using CBCT shows the analysis made by LCA can be reliable.

Our findings suggest that the LCA might provide important details regarding the anatomy and severity of airway obstructions. According to research, there is a moderate association between airway area and volume measured by CBCT and those measured by lateral cephalogram (r = 0.86). More volume is contained in a broader area [15]. A strong relationship between velopharyngeal area and volume was also shown in Lenza et al.'s investigation [17]. Long-term changes in airway function may affect face shape. Most patients with greater anterior face height also had nasopharyngeal dysfunction, suggesting that the relationship between airway function and facial morphology is more complex than previously thought. This could potentially impact both the health and aesthetic aspects of your dental condition [3, 23-27]. In patients who are known or believed to be difficult for intubation, this provides a baseline for the anesthesiologist's ability to control the airway. Clinical methods like history and examination may not be as helpful as a lateral head film in certain situations.

The lateral cephalogram has no cross-sectional area or volume values, which is a significant flaw. Recently, there has been increased interest in using CBCT technology as a diagnostic instrument to examine the airways [20]. Zooming in poses no issues because the 3D model is recreated from the raw data. Measurements may be made precisely and anatomically correct thanks to the isotropic image that the CBCT creates. Since CBCT technology is less readily available than traditional radiography, orthodontic patients are not frequently referred for the test [21]. Enciso et al.'s study [9] using cone-beam CT revealed that patients with OSA had lower UAS cross-sectional areas and lower lateral dimensions than snorers. According to Guilleminault et al.'s research, OSA recurrence in previously treated adolescent patients was associated with a significant constriction of the posterior airway space [10]. Other UAS soft tissue characteristics that have been suggested as significant morphological determinants in OSA include the length of the soft palate, the size of the tongue, and the adenoids [11, 12].

In our research, the mean value of volume evaluated in 3D CBCT in males was 4198±1008 mm^3^, while it was 2980±1134.5 mm^3^. The observations were found to have a positive correlation with carrying out statistical analysis with an increased volume of pharyngeal space in males as compared to that of females (p=0.006). The area of pharyngeal space calculated using LCA in males was 370.1±60.9 mm^2^, while it was 301.9±88 mm^2^ in females. The observations were found to have a positive correlation with carrying out statistical analysis with an increased area of pharyngeal space in males as compared to that of females (p=0.005). During the statistical analysis, these observations were found to have a positive correlation with increased volume of pharyngeal space in males as compared to that of females (p=0.006). The evaluation using LCA was considered reliable (p=0.001).

The potential link between aberrant breathing patterns and craniofacial development has received a lot of attention in the scholarly literature [14, 15]. Development, growth, respiration, speech, and neuromuscular responses are all significantly impacted by airway function [16, 17]. OSA is also hypothesized to be influenced by the size of the upper airway [18]. This is crucial for teenagers who are exhibiting skeletal abnormalities and abnormal facial signs. According to research, many occurrences of malocclusion, sometimes known as "adenoid face," can be linked to breathing issues. Other studies of the upper airway have looked into various skeletal patterns [19, 20].

Even though airway blockages may resolve naturally over time, it is ideal to study the effects of airway obstructions on dentition, speech, and craniofacial development through experimental examinations. As a result, orthodontists require trustworthy diagnostic tools that adequately inform them, as well as any other medical specialists who might need to be consulted [21]. A lateral cephalogram is frequently used by the orthodontist to provide limited, subjective examinations of suspected airway abnormalities [22-24]. This type of airway anatomy investigation has limited diagnostic utility, though. When trying to depict a three-dimensional structure in a two-dimensional format, issues such as distortion, differences in magnification, and the superimposition of the bilateral craniofacial components can occur [17-19]. Numerous studies have examined the relationship between UAS size and other cranial characteristics using lateral cephalograms, computed tomography, or cone-beam CT scans in individuals who are generally healthy and free of pharyngeal or breathing issues [9-15].

It is debatable whether there is a relationship between UAS dimensions and sagittal skeletal patterns because different results have been recorded. In certain investigations [16-18], it has been demonstrated that sagittal skeletal malocclusion affects UAS size, while in other studies [20-21], no association has been demonstrated. Nasal cavity, oral cavity, and hypopharynx), clashing due to correlated elements such as vertical and horizontal growth patterns and measuring area [12, 14, 20], categorization based on skeleton vs. teeth, requirements for inclusion (overweight and smoking), etc. [20, 21], etc., may all be factors that contribute to the differences seen in the aforementioned research. There was a strong connection between lateral cephalograms and CBCT pictures in the current research; however, CBCT technology allows for the determination of true distances and angles, which are not evident in 2D cephalograms. which aids in the accurate evaluation of craniofacial growth and development [26]. For instance, clinical malocclusion manifests itself in three dimensions, yet when treating it, clinicians often just look at the condition from the anteroposterior rather than also considering the vertical and transverse dimensions. Therefore, a 3D malocclusion description is possible using CBCT pictures in addition to a 3D airway evaluation.

This study's lack of functional testing precludes the establishment of a threshold for demonstrating the degree to which airway volume may impair function. More research is required to answer this issue. Airway channel size and shape have been linked to increased airflow resistance, according to some research [14, 27]. In addition, 3D airway examination may aid in distinguishing the pharyngeal airway form and providing a more accurate diagnosis of airway issues.

## Conclusions

This preliminary study shows that the area estimated for the pharyngeal airway on LCA correlates strongly with the volume determined by a CBCT scan. Since we have considered pharyngeal space analysis using CBCT to be a reliable and standard methodology, therefore positive correlation of area calculated using LCA with volume calculated using CBCT shows the analysis made by LCA can be reliable. In carrying out statistical analysis, the values of the area of pharyngeal space in different sexes were calculated using LCA than by CBCT. The evaluation using LCA was considered reliable. To learn how airway volume may impact function, further research incorporating functional testing is needed.
